# Brainstem Anesthesia and Cardiac Arrest Following Peribulbar Block: A Case Report and Systematic Review of the Literature

**DOI:** 10.3390/jcm13216572

**Published:** 2024-11-01

**Authors:** Matteo Ripa, Chiara Schipa, Paola Aceto, Goutham Kanikaram, Neeraj Apoorva Shah

**Affiliations:** 1 Department of Ophthalmology, Sankara Eye Hospital, Jaipur 302039, Rajasthan, India; 2 Catholic University “Sacro Cuore”, 00168 Rome, Lazio, Italy; 3 Department of Emergency, Anesthesiological and Reanimation Sciences, Fondazione Policlinico Universitario A. Gemelli IRCCS, 00168 Rome, Lazio, Italy

**Keywords:** peribulbar block, ocular anesthesia, brainstem anesthesia, cardiac arrest

## Abstract

**Background**: We report a case of brainstem anesthesia (BSA) and subsequent cardiac arrest following a routinary peribulbar block (PB) in a patient scheduled for cataract extraction and intraocular lens (IOL) implantation, thus providing a reference for further analysis of this potentially catastrophic life-threatening complication and to evaluate the current knowledge in terms of incidence, physiopathology management, and treatment of the BSA following PB. **Methods**: Three databases (PubMed, Embase, and Scopus) were checked to perform a systematic review of all available studies in the English Language following the Preferred Reporting Items for Systematic Reviews and Meta-Analyses (PRISMA) guidelines to evaluate relevant studies that clearly described BSA following BSA. **Results**: Our literature search identified 15 cases. All the patients experienced BSA-related symptoms, including respiratory arrest, seizure, heart rate, and blood pressure abnormalities. All the patients with respiratory arrest required assisted ventilation with intubation, whereas patients with seizures were administered intravenous midazolam. Only one patient experienced cardiac arrest and underwent cardiac resuscitation. Surgery was aborted and deferred in 5 out of 15 patients, whereas 4 out of 15 underwent surgery after extubation. **Conclusions**: Despite the BSA incidence after the PB being very low, this possible life-threatening rare event should be considered in patients losing their consciousness and becoming apneic after the block. Therefore, prompt recognition and immediate treatment are paramount to cope with this potentially catastrophic scenario and save the patient’s life.

## 1. Introduction

Over the years, different regional anesthetic techniques, including topical, retrobulbar, peribulbar, and sub-Tenon blocks, have been widely adopted in patients undergoing ocular surgery to provide akinesia and analgesia [1]. Nowadays, peribulbar block (PB) remains the most commonly used technique in patients undergoing cataract extraction and several ocular procedures such as corneal transplant, glaucoma, and vitreoretinal procedures [1,2,3]. According to the current literature, the major complication rate following PB in patients undergoing ocular surgery was 0.006% [4]. Despite the higher safety profile and lower incidence complication rate than the retrobulbar block (RB), PB has a slower onset of analgesia and often requires larger volumes of anesthetic agents and multiple injections [5]. Nonetheless, despite being rare, PB may have potentially life-threatening complications such as cerebrovascular accidents, seizures, brainstem anesthesia (BSA), respiratory depression, and cardiac arrest [6,7,8,9,10,11,12,13,14,15,16]. The central nervous system (CNS) involvement has been rarely reported following PB block. In 1988, Hamilton et al. reported only one case of CNS spread in 6800 (0.015%) extraconal (true peribulbar) blocks using a 38 mm needle [17]. According to the 2007 United Kingdom National Health Service Survey, out of 115.700 PB blocks, eight patients who underwent PB experienced “potentially life-threatening” complications (0.7 per 10,000), most consistent with BSA [16]. In 2013, Chin et al. reported that the estimated incidence of BSA following PB was 0.020% [18]. So far, less than 20 BSA cases following PB have been reported. Herein, we systematically review the literature to evaluate the current knowledge in terms of incidence, physiopathology management, and treatment of the BSA following PB and report a case of BSA and subsequent cardiac arrest following a routinary PB block in a patient scheduled for cataract extraction and intraocular lens implantation, thus providing a reference for further analysis of this potentially catastrophic life-threatening complication.

## 2. Case Report

An 82-year-old Indian man with bilateral cataracts recruited from a cataract screening camp was admitted to our hospital to undergo lens extraction using the small incision cataract surgery technique combined with implantation of an intraocular lens in the left eye under PB anesthesia. He was hemodynamically stable in the screening camp examination, and no comorbid conditions were reported. At the hospital admission, he was neither hypertensive nor hyper/hypoglycemic, and the hospital pre-anesthesiological assessment was unremarkable. The ophthalmic examination revealed an axial length of 23.73 mm in the right and 23.54 mm in the left eye. The intraocular pressure was 12 mmHg in the right and 14 mmHg in the left eye. Before the peribulbar block, two drops of proparacaine hydrochloride 0.5% (Paracain, Sunways Pvt. Ltd., Mumbai, India) and two drops of a commercially available mydriatic mixture (0.8% tropicamide + 5% phenylephrine, Tropicacyl plus, Sunways Pvt. Ltd., Mumbai, India) were instilled 15 min preoperatively in the holding area by the ophthalmologist in the left eye. Once shifted to the block room, the patient underwent standard monitoring, including pulse oximetry and non-invasive blood pressure. The pre-anesthesiologic vital signs were within normal limits [blood pressure (BP): 126/68 mmHg, oxygen saturation (SpO_2_): 98%, and pulse: 90/min] and the patient (American Society of Anaesthesiologists) ASA’s (American Society of Anaesthesiologists) grade was 2. Following the vital signs check and one drop of proparacaine hydrochloride 0.5% (Paracain, Sunways Pvt. Ltd., India) in both eyes, the peri-orbital skin was cleansed with 5% povidone-iodine for 5 min.

Soon after, the PB was performed by an experienced ophthalmologist with more than 2500 ocular block experiences (M.R.). It consisted of a mixture of 5 mL of commercially available 2% lignocaine hydrochloride with 1:200,000 epinephrine (Lox 2%, Neon Laboratories Ltd., Mumbai, India) and 50 U·mL^−1^ hyaluronidase placed in a 5 mL syringe.

Using a 25 mm 24 gauge (G) sharp bevel disposable needle, the initial injection was administered at the inferotemporal orbital rim, midway between the lateral canthus and lateral limbus, with the eye fixed in its primary position. Following negative aspiration and ‘Tethering test’, the initial 5 mL of the local anesthetic mixture was slowly injected with no resistance. After 2 min, additional medial canthus anesthesia was provided. Specifically, the 24 G needle was placed into the medial canthus to its full depth in a vertical direction, and neither contact with the bone nor resistance during the needle’s insertion was noted. After a negative aspiration, a 5 mL mixture of 2% lignocaine hydrochloride, 1:200,000 epinephrine (Lox 2%, Neon Laboratories Ltd., Mumbai, India), and 50 U·mL^−1^ hyaluronidase was slowly administered. Throughout the procedure, the patient maintained a neutral gaze. The ‘Tethering test’ was not undertaken. After withdrawing the needle, total akinesia of the globe and lids was achieved.

The patient laid on the block room bed for 5 min before being shifted to the operatory theatre. During the shifting, the patient stumbled and fell over the floor, becoming immediately unresponsive to verbal stimuli. His breathing became irregular, he rapidly became apneic and went into complete cardiac arrest. The carotid pulse was immediately checked for 5 s. The cardiac monitor showed pulseless electrical activity (PEA) arrest, immediate cardiac resuscitation (CPR) was performed, and the PEA advanced cardiac life support (ACLS) algorithm followed. During resuscitation, the patient required assisted breathing with an Ambu bag containing 100% oxygen. During the CPR, the patient received three doses of 1 mg of adrenaline. The return of spontaneous circulation (ROSC) was achieved after 15 min. Following the ROSC, the patient regained consciousness, and the twelve-lead electrocardiograms revealed sinus tachycardia without ST-segment elevation. The patient’s BP was 140/110 mmHg, SpO_2_: 98%, and HR 104 pulse/min. No signs of anemia or external bleeding were observed. Therefore, the planned surgery was aborted, and the patient was transferred to another tertiary hospital to receive intensive care unit (ICU) care and further evaluation and management.

## 3. Materials and Methods

### 3.1. Eligibility Criteria

To evaluate the current knowledge in terms of incidence, physiopathology management, and treatment of the BSA following PB, we conducted a systematic review according to the Preferred Reporting for Systematic Reviews and Meta-Analyses (PRISMA) guidelines [19]. We registered our research with the International Prospective Register of Systematic Reviews (PROSPERO) database (identifier: CRD42024554485). All papers, such as case reports, retrospective and prospective case series, and comparative studies reporting BSA following PB in patients scheduled for ocular surgery, were included. Our analysis excluded literature review studies, conference abstracts, theses and dissertations, and book chapters. Articles that reported incomplete data were also excluded. In addition, all papers referring to BMA following RB were excluded from our analysis.

### 3.2. Search Methods

PubMed, Embase, and Scopus databases were checked from inception until 1 June 2024, using free text to evaluate current knowledge regarding the incidence, physiopathology management, and treatment of the BSA following PB in patients scheduled for ocular surgery. The keywords were identified based on readings relevant to the study’s content. Specifically, the keywords were combined with boolean operators such as OR and AND to broaden, focus, and restrict the search. Furthermore, the search employed known and extended vocabulary without database filters. Our primary search words were “Brainstem Anesthesia” AND “Peribulbar block” OR “Peribulbar Anesthesia”. This proceeded until adding more terms yielded no new results. In addition, we hand-searched the bibliographies of included publications to find additional research that was not retrieved during the initial database search. Supplementary Materials S1 and S2 report the detailed search strategy and Preferred Reporting Items for Systematic Reviews and Meta-Analyses (PRISMA) Checklist.

### 3.3. Study Selection and Data Collection

Two investigators (M.R. and G.K.) independently investigated study titles and abstracts to identify articles evaluating current knowledge regarding the incidence, physiopathology management, and therapy of BSA following PB in patients scheduled for ocular surgery. In addition, the two reviewers (M.R. and G.K.) independently evaluated the entire text of the remaining studies to evaluate the research design. Studies with research designs that did not match the inclusion criteria were excluded. Furthermore, the two investigators separately retrieved baseline and outcomes data and reviewed discrepancies for adjudication if consensus was not established with a third author (P.A.). The reasons for exclusion were documented. We extracted the following data from each article: first author, country, study design, total sample size, number of patients with BSA, age, gender, eye, axial length, co-morbidities, ASA (American Society of Anaesthesiologists) grade, PB mixture, needle used (length and thickness), number and site of injection, time between PB and symptoms onset, symptoms, management, and outcome. We used the Covidence systematic review software © (Veritas Health Innovation, Melbourne, Australia), available at www.covidence.org [19], for extraction purposes until 15 June 2024.

### 3.4. Risk of Bias Assessment

Two authors (M.R. and G.K.) independently appraised the methodological quality of each study by using the Newcastle–Ottawa Scale (NOS) [20] for the observational study and the Joanna Briggs Institute (JBI) Critical Appraisal Checklist for Case Reports, which consists of eight yes/no/unclear questions. Quality assessment data individually appraised by each of the reviewers were compared. M.R. and G.K. discussed the discrepancies with a third author (P.A.) for adjudication if consensus could not be achieved. Two independent researchers (M.R. and G.K.) separately applied the Grades of Recommendation, Assessment, Development, and Evaluation (GRADE) criteria for risk of bias evaluations. A third author (P.A.) was employed in case of doubts or no consensus. There were four categories for the evidence: high, moderate, low, and extremely low. Based on the factors that reduce the quality of the evidence—the risk of bias, inconsistency, indirect evidence, inaccuracy and publication bias, a large effect size, the dose–response gradient, and residual confounders—the primary conclusions from the synthesis of the included studies were presented [21].

## 4. Results

### 4.1. Study Selection

**Figure 1:**
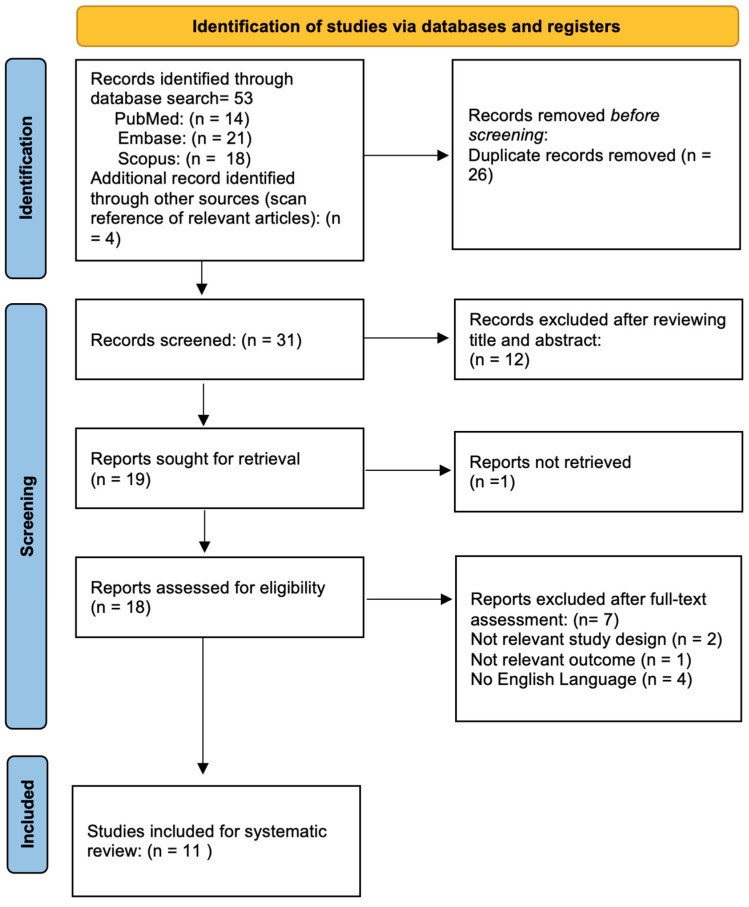
Flow diagram of the study selection process.

Figure 1 illustrates the flow chart of our analysis selection and identification process. The search yielded 53 indexed articles (14, 21, and 18 records from PubMed, Embase, and Scopus, respectively). A search of the reference list yielded 4 other articles for a total of 57 articles retrieved. After duplication removal, we screened a total of 31 articles. After the title and abstract screening, we excluded 12 studies, and only 18 full-text studies were retrieved and assessed for final eligibility. Only one study could not have been retrieved. Furthermore, an additional eight articles were excluded either due to the language, wrong outcome, or inappropriate study design. Finally, a total of 11 articles met the inclusion and exclusion criteria and were included in the systematic review [6,7,8,9,10,11,12,13,14,15,16].

### 4.2. Study Characteristics

A summary of the main characteristics, including first author, country, study design, total sample size, number of patients with BSA, age, gender, eye, axial length, co-morbidities, ASA (American Society of Anaesthesiologists) grade, PB mixture, needle used (length and thickness), number and site of injection, time between PB and symptoms onset, symptoms, management, and outcome are summarized in Table 1. We assessed one cross-sectional study [16] and ten case reports covering the last 30 years of the literature [6,7,8,9,10,11,12,13,14,15]. Indeed, the included articles were published between 2 April 1995 [15] and 29 January 2021 [6]. Fifteen patients experienced BSA following PB; among them, two patients received two consecutive PB injections, initially placed in the inferotemporal junction of the medial two-thirds and the lateral third of the lower eyelid, and afterward either in the superior junction of the medial third and the lateral two-thirds of the superior orbital margin or at the medial conal space, respectively [7,15]. The patients’ ages experiencing BSA ranged from 42 to 83. Overall, three studies involved patients from European countries [9,10,16] three involved patients from American countries [11,13,14] and five involved patients from Asian countries [6,7,8,12,15]. The most common mixture of LA contained lignocaine (mixture of lidocaine 2%, with hyaluronidase 50 U·mL^−1^, and adrenaline 1:200,000) as reported in six out of eleven studies [6,7,8,9,12,15], whereas the use of lidocaine 1.0% and bupivacaine 0.5%, lidocaine 2% alone or combined with ropivacaine 1% were reported in four studies, respectively [10,11,13,14]. Only one study did not report the LA mixture used [16]. The needle thickness ranged from 22 to 27 G, whereas the most widely used needle length was 25 mm. Despite Edge et al. using a 34 mm needle, only 24 mm were advanced in the peribulbar space [15]. In contrast, Palte et al. used a 27 G needle with a 31 mm length [11]. Regarding systemic comorbidities, 6 out of 15 patients had a history of hypertension [7,9,10,11,13,16], and 3 out of 15 were diabetic [9,11,15]. Only five studies reported an ASA score that ranged from 2 to 3 [7,10,13,14,15], with only one patient having an ASA score equal to 3 [15]. All the patients experienced BSA-related symptoms, including respiratory arrest (8 out of 15) [6,7,9,10,13,14,15], seizure (2 out of 15) [6,8], and heart rate (2 out of 15) (tachycardia/bradycardia) [7,8] and blood pressure abnormalities (3 out of 15) (hypotension/hypertension) [6,7,14] until deafness and nerve palsy within 20 min after the PB (ranging from a few seconds to 20 min after the PB) [11,12]. All the patients with respiratory arrest required assisted ventilation with intubation [6,7,9,10,13,14,15], whereas patients with seizures were administered IV (intravenous) midazolam [6,8]. Only one patient experienced cardiac arrest and underwent cardiac resuscitation [10]. In total, 5 out of 15 patients [6,8,9,10,11] aborted and deferred surgery, whereas 4 out of 15 underwent surgery either after extubation or under general anesthesia [7,13,14,15] Data regarding surgery in 5 out of 15 patients were not reported [16]. One patient underwent BSA with contralateral third nerve palsy after surgery, and total recovery was achieved 2 h postoperatively [12].

### 4.3. Risk of Bias

Tables S1 and S2, available in Supplementary Material S3, summarise all studies’ risk of bias evaluation. The quality rating of the cross-sectional study was 8 out of the maximum score on the Newcastle–Ottawa Scale [16]. According to the JBI Critical Appraisal Checklist for Case Reports, the overall quality of the cases was good, as most articles were determined to have a low risk of bias (7 out of the maximum score) [6,7,8,9,10,11,12,13,14,15]. The quality of the evidence was low according to the GRADE assessment due to the study’s design of the included articles (10 out of 11 were case reports) (Table S3, available in Supplementary Material S3).

## 5. Discussion

To the best of our knowledge, less than 20 cases of BSA following PB have been reported in the literature among patients undergoing ocular surgery. Herein, we reported a rare case of BSA and subsequent cardiac arrest following a routinary PB block in a patient scheduled for ocular surgery.

BSA is a rare, potentially life-threatening complication of regional ophthalmic anesthesia that occurs when an injected anesthetic agent directly enters the subarachnoid space or a local anesthetic agent spreads into the central nervous system, leading to warning non-life-threatening symptoms such as amaurosis, deafness, aphasia, twitching, slurred speech till more severe signs such as respiratory depression, cardiovascular impairment, and cardio-respiratory arrest [8,10,11]. All the signs and symptoms of CNS involvement after inadvertent anesthetic injection are related to several factors, such as the anesthetic agent dose and concentration, the depth of needle insertion, and the force and time used during the injection. Despite its higher incidence associated with RBs, less than 20 cases of BSA following PB have been reported in the literature in patients undergoing ocular surgery. Nonetheless, the incidence of BSA following different ocular anesthesia techniques, including RB, PB, and sub-Tenon’s block, is variable. According to Nicoll et al., who evaluated 6000 consecutive patients undergoing RB [22], the incidence of BSA following RB was 0.27%. In contrast, Rapati et al. reported a BSA incidence of 0.044% in 4500 subsequent cases of RB studied from 1981 to 1990 [23]. BSA following PB occurs at a rate of 0.02%. The literature describes a few cases of BSA following sub-Tenon’s blocks, which were probably triggered by a dural rupture during scissor dissection and/or central spread of local anesthetic following blunt-needle sub-Tenon’s block [24,25,26]. The subarachnoid spread of local anesthetic following ocular anesthesia may be related to several mechanisms, including the accidental intravascular injection of an anesthetic agent or the needle’s direct neural breach of the optic nerve. Despite the aspiration being always performed before the injection of local anesthetic to avoid a direct intravascular spread, an accidental puncture of the orbital artery may cause the retrograde flow of anesthetic from the orbital artery to the internal carotid artery, thus directly affecting the thalamus and other midbrain structures [14]. The intra-arterial injection immediately determines grand mal seizure activity associated with respiratory arrest and hemodynamic changes [27]. BSA following local anesthetic penetration of the dural sheath of the intraorbital optic nerve with resultant subdural or subarachnoid spread involving the respiratory center and chiasmatic cistern has been widely investigated. In 1969, Reed et al. showed the presence of the previously injected radiopaque dye within the intracranial subdural space after an orbitography on a patient with an intraorbital neoplasia [28]. In 1984, Drysdale showed that when radio-opaque dye is injected into the intraorbital subdural space, the dye is found in the midbrain around the respiratory center [29]. A few years later, Wang et al. found that the intrasheath injection of methylene blue could have been identified in the subarachnoid space of the optic nerve sheath and into the chiasmatic cistern in the middle cranial fossa [30]. Direct contact with the optic nerve sheath is more likely to happen when longer needles, such as those used in RB, are placed into the extraconal space via the inferotemporal route, as reported by Edge and Perils [15]. Nonetheless, BSA following PB has been reported in blocks performed with shorter needles (e.g., 25 mm), suggesting that the needle’s angle and trajectory play a paramount role compared to the absolute length of the needles [6,7,8,9,10,13,14]. The doctor’s position may also play a role in the contact between the advancing needle tip and the optic nerve during the block. Indeed, in the head-end position, despite the PB being performed in a neutral gaze, patients tend to stare at the doctor. The patient’s upward gaze exposes the optic nerve toward the advancing needle tip, thus enhancing the risk of accidental puncture. Anatomical studies have shown a higher presence of arachnoid villi in the optic nerve, which either lies within the meningeal dural sheath or protrudes through it. These exposed villi may represent the route through which the anesthetic agent can be absorbed and then spread to the contiguous subarachnoid spaces, reaching the medullary centers. Therefore, the manual compression that allows the spread of the anesthetic agent may also have determined its absorption by the arachnoid villi and its direct spread to cerebral structures [6]. In our case, the needle pathway, as the blocker position, may have determined accidental contact with the optic nerve sheath. Regardless of the technique, the onset of BSA-related symptoms may range from 2 to 20 min after the block. In our case, the patient underwent respiratory arrest and subsequent cardiac arrest within 5 min of the block. Our case is consistent with the report of Basu et al. and Kazancıoğlu et al., whose patients underwent respiratory arrest and loss of consciousness in 5 and 7 min, respectively [6,10]. As per our case, the quick onset of the symptoms, the fast recovery, and the lack of severe cardiovascular condition supported the diagnosis of BSA. We could rule out the accidental intravascular anesthetic injection as the test aspiration before the procedure was negative as there was no periocular hematoma pointing to any vascular breach, and neither CNS excitatory symptoms nor seizure was present. In addition, taking into account that the 2% lidocaine mixed with vasoconstrictors such as epinephrine 1:200,000 toxic dose is 7 mg/kg, we could also exclude lidocaine toxicity as we injected only 10 mL of anesthetic. Therefore, the sequence of events and their timing points, the clinical course, and complete recovery with no remaining visual or neurological defects supported the diagnosis of brainstem anesthesia. Nowadays, there is still no consensus on whether the surgery should be aborted. According to the retrieved studies, respiratory arrest was a common clinical finding in 7 out of 15 patients requiring intubation and mechanical ventilation, but only 4 out of 15 underwent surgery either after extubation or under general anesthesia [7,13,14,15] Nonetheless, proceeding with elective ocular surgeries, such as lens extraction, in a patient who has experienced a significant complication like BSA may entail substantial risks, making it essential to exercise caution in such cases. To the best of our knowledge, only one case of cardiac arrest following PB has been previously described. In 2017, Kazancıoğlu et al. reported a case of cataract surgery that received PB with 6 mL of 2% lidocaine hydrochloride. Following the injection, cardiac arrest occurred. The patient was intubated and mechanically ventilated for 30 min, and CPR and 1 mg of adrenalin were applied to the patient. Finally, the patient regained consciousness, was extubated, and transferred to the ICU for further follow-up [10]. Despite these catastrophic presentations, a few reports suggested an initial cardiovascular stimulation, with an increase in pulse and blood pressure. This sympathetic overactivity is suggestive of a cephalic entrance of local anesthetic. Indeed, anesthetic entry via this route suppresses the depressor function ahead of the excitatory centers. Blockage of the glossopharyngeal nerve suppresses the carotid sinus reflex, causing tachycardia in the context of hypertension [9]. Nonetheless, Palte et al. reported an atypical presentation of BSA. The patient did not experience loss of consciousness, respiratory depression, hypotension, contralateral ophthalmoplegia, or amaurosis. Transient malignant hypertension, hearing loss, and slurred speech were the only presented symptoms. The atypical presentation was triggered by needle penetration of the lateral segment of the dural sheath, which abolished parasympathetic efferent activity and overexpressed sympathetic activity. This led to a hypertensive crisis as the anesthetic reached the subdural plane over the lateral optic chiasm and hypothalamus [11]. Regardless of the etiology, early diagnosis and supportive care are critical to the patient’s survival once BSA develops [31]. Indeed, BSA symptoms onset is up to 20 min after the PB is administered, and recovery begins 60 to 90 min later. Since there might be aftereffects for a few hours, the patient must be closely monitored in the critical care unit. The most suitable course of treatment varies according to the symptoms. It may involve the following: inotropic circulatory support, benzodiazepine, or barbiturate usage for seizure control, intubation, and mechanical ventilation in the event of respiratory arrest. Supportive treatment should be used, and vital signs and oxygen saturation should be maintained until recovery. In critical situations, the patient could require CPR and airway management [6,7,8,9,10,11,12,13,14,15]. Prevention is paramount to avoid complications following PB, regardless of the mechanism [32]. Indeed, even though BSA is a rare event, everyone should be ready to handle its consequences and face this potentially life-threatening emergency clinical scenario. Therefore, an anesthesiologist must be on the scene during locoregional anesthesia in patients undergoing ophthalmic surgery, regardless of who performs the blockade, to guarantee the patient’s safety. Furthermore, the monitoring protocols for ophthalmic local anesthesia should be analogous to those followed for general anesthesia, and the operating room ought to be equipped with all the necessary technical and human resources to deal with any complications that may arise after locoregional anesthesia [33]. Beyond the anesthesiological preventive measures, other prevention elements should always be clarified before locoregional anesthesia in patients undergoing ophthalmic surgery. The systematic practice of needle test aspiration before each injection [34], the “tethering test” [35], maintaining the globe in a neutral gaze till the completion of the PB, and the use of needles no longer than 25 mm should always be considered before any ocular block. Despite the use of ultrasound in ophthalmic surgery reducing the rate of complications and improving the safety and performance of these techniques, it is quite difficult to routinely adopt it, especially in high-volume ocular surgery centers performing more than 100 surgeries per day [36]. To the best of our knowledge, no systematic reviews analyzing all the current literature regarding the occurrence of BSA following PB have been published. Despite this strength, this case report and the systematic review have several limitations. First, most of the included studies were case reports whose study design represents a bias per se. Therefore, case reports have provided more evidence due to the event’s rarity. Second, we could not retrieve radiological imaging or cardiology and neuro-medicine consultation reports following cardiac arrest in our patient. The absence of orbit imaging could not provide us with any anatomical information that could characterize the patient at a greater risk of developing complications during local ocular anesthesia. Third, we could not determine whether the needles referenced in the retrieved literature studies were inserted to their entire lengths. While the angle, trajectory, and length of the needles are essential factors in peribulbar anesthesia, the depth of needle insertion may play a significant role in the onset of BSA. The absence of detailed information regarding the needles’ insertion depths in the reviewed studies hinders our ability to evaluate their potential impact on clinical outcomes and limits the broader applicability of our findings. Finally, we could not perform a meta-analytical analysis to evaluate the BSA prevalence following PB due to the absence of data. Therefore, cross-sectional or longitudinal studies reporting the prevalence of BSA following PB are advised.

## 6. Conclusions

Despite the BSA incidence after the PB being very low, this possible life-threatening rare event should be considered in patients losing their consciousness and becoming apneic up to 20 min after the block. Therefore, prompt recognition and supportive treatments are paramount to cope with this scenario and save the patient’s life. The presence of an anesthesiologist during ocular blocks is necessary to ensure the patient’s safety. All operatory and block rooms should be equipped with basic resuscitation instruments, and every patient during ophthalmic local anesthesia should be monitored as those undergoing general anesthesia. The PB should be performed by an experienced surgeon, taking all the precautions. All technical and human resources should be available in the operating room to deal with complications following locoregional anesthesia. Finally, being aware of this potentially catastrophic complication that can lead to cardiac arrest and how to deal with it could save the lives of patients undergoing ophthalmic surgeries.

## Figures and Tables

**Table 1:** Characteristics of studies included in the systematic review.

First Author	Country	Study Design	Total Sample Size (n.) Number of Patients with BSA (n. and %)	Age (Years) Gender (n. and %)	Eye	Axial Lenght	Co-Morbidities ASA Grade	PB Mixture Needle Used (Length and Thickness) N. of Injection Site of Injection	Time Between PB and Symptoms Onset	Symptoms	Management	Outcome
Edge et al., 1995 [15]	Saudi Arabia	Case Report	1 1 (100%)	69 Male	Left	25.90 mm	Asthma Chronic joint pains DM ASA: 3	6.0 mixture of bupivacaine 0.5%, lignocaine 2.0% and 100 IU hyaluronidase 37 mm 25 G 2 Injection 1: inferotemporal, transcutaneous injection inserted at the junction of the medial two-thirds and the lateral third of the lower eyelid Injection 2: superiorly at the junction of the medial third and the lateral two-thirds of the superior orbital margin	20 min	Respiratory Arrest	Respiratory Arrest: Ventilatory assistance: Intubation and Mask	Extubation and Surgery Performed
Gomez et al., 1997 [14]	Brazil	Case Report	1 1 (100%)	75 Female	Right	N/a	Coronary artery disease ASA: 2	10 mL of 50/50 mixture of lidocaine 1.0% and bupivacaine 0.5% with 50 U·mL^−1^ hyaluronidase 25 mm, 22 G 1 Inferotemporal, transcutaneous injection inserted at the junction of the medial two-thirds and the lateral third of the lower eyelid	Few seconds after the PB	Respiratory Arrest Hypotension and bradycardia	Respiratory Arrest: Ventilatory assistance: Intubation and Mask Bradicardia: Atropine (1.0 mg) Hypotension: Ephedrine (5 mg)	Extubation and Surgery Performed
Eke et al., 2007 [16]	UK	Cross-sectional Study	117.700 5 (0.0005%)	Patient 1: 82 Patient 2: 77 Patient 3: 83 Patient 4: 70 Patient 5: 73 Patient 1: N/a Patient 2: N/a Patient 3: N/a Patient 4: N/a Patient 5: N/a	N/a	N/a	Patient 1: HTN Patient 2: Ischaemic heart disease Patient 3: Angina; Patient 4: Ischaemic heart disease Patient 5: Osteoarthritis ASA Patient 1: N/a ASA Patient 2: N/a ASA Patient 3: N/a ASA Patient 4: N/a ASA Patient 5: N/a	N/a N/a N/a N/a	Patient 1: 5 min after PB Patient 2: N/a Patient 3: 2 min after PB Patient 4: 5 min after PB Patient 5: 1 min after PB	Patient 1: Grand mal fit Patient 2: Grand mal fit Patient 3: Apnea for 10 min, Patient 4: numb legs Patient 5: Reduced oxygen saturation	Patient 1: N/a Patient 2: N/a Patient 3: Transferred to medical ward Patient 4: N/a Patient 5: Transferred to medical ward	Patient 1: no long-term effects Patient 2: no long-term effects Patient 3: no long-term effects Patient 4: no long-term effects Patient 5: no long-term effects
Carneiro et al., 2007 [13]	Brazil	Case Report	1 1 (100%)	60 Female	Right	22.71 mm	HTN ASA: 2	Mixture of: 1 mL of lidocaine, 4 mL of 0.75% bupivacaine and 20 U·mL^−1^ of hyaluronidase 25 mm, 25 G 1 Inferotemporal, transcutaneous injection inserted at the junction of the medial two-thirds and the lateral third of the lower eyelid	Few seconds after the PB	Respiratory Arrest Lost of Consciousness	Respiratory Arrest: Ventilatory assistance: Manual ventilation with a face mask and 100% oxygen, intubation General Anaesthesia Induction: A bolus of intravenous propofol (70 mg) and isoflurane	30 min after intubation: limbs movement Surgery performed under GA
Jaichandran et al., 2013 [12]	India	Case Report	1 1 (100%)	60 Male	Right	25 mm	None ASA: N/a	5 mL mixture of of 2% lignocaine solution (and of 0.5% bupivacaine solution along with hyaluronidase 25 IU/mL N/a 1 Inferotemporal, transcutaneous injection inserted at the junction of the medial two-thirds and the lateral third of the lower eyelid	N/a	Contralateral third nerve palsy	None: Spontaneous Recovery	Uneventful Surgery, Contralateral third nerve palsy recovered after 2 h postoperatively
Palte et al., 2017 [11]	USA	Case Report	1 1 (100%)	42 Male	Left	N/a	HTN and DM ASA: N/a	9 mL mixture containing lidocaine 2%, ropivacaine 1%, and Hylenex 7.5 IU/mL 31mm, 27 G 1 Inferotemporal, transconjunctival injection inserted at the junction of the medial two-thirds and the lateral third of the lower eyelid	10 min	Paroxysmal tachycardia (140/min) Acute, severe HTN (240/140 mmHg) Deafness	Paroxysmal tachycardia: Intravenous nicardipine (200 mcg) HTN: labetalol (10 mg) Deafness: None	Surgery Aborted Patient transferred to a tertiary center for an urgent CT scan to exclude stroke
Kazancıoğlu, 2017 [10]	Turkey	Case Report	1 1 (100%)	68 Female	Right	N/a	HTN ASA: 2	6 mL of 2% lidocaine hydrochloride 25 mm, 25 G 1 Middle of the lateral limbus and lateral canthus at the inferotemporal lower orbital rim of the lower eyelid	10–15 min after PB	Cardiac Arrest	Respiratory Arrest: Ventilatory assistance: Intubation Circulation: CPR and 1 mg adrenalin	Surgery Aborted Extubated after 30 min and shifted to ICU
Vohra et al., 2019 [9]	UK	Case Report	1 1 (100%)	52 Female	Left	21.86 mm	HTN and DM ASA: N/a	Mixture of 3 mL of plain 2% lignocaine and 3mL of 0.75% levobupivacaine with 300 units of hyaluronidase 25 mm; 25 G 1 Medial canthus in vertical direction	Few seconds after the PB	Respiratory Arrest Supraventricular tachycardia (HR 150 bpm) HTN (185/130 mm Hg)	Respiratory Arrest: Ventilatory assistance: Intubation Supraventricular tachycardia and HTN: IV Labetalol (10 mg), Acetazolamide (500 mg) and 50 mL of 20% Mannitol	Surgery Aborted
Tayab et al., 2019 [8]	India	Case Report	1 1 (100%)	70 Male	Left	N/a	None ASA: N/a	8 mL mixture of 1% lignocaine and 0.5% bupivacaine with 50 IU/mL Hyaluronidase 25 mm, 24 G 1 Inferotemporal, transconjunctival injection inserted at the junction of the medial two-thirds and the lateral third of the lower eyelid	Few minutes after the PB	Tachycardia (150 beats/min) HTN (190/120 mmHg) Seizure	Respiratory Arrest: Ventilatory assistance: Intubation Tachycardia: Esmolol (80 mg) Seizure: IV midazolam (2.0 mg)	Surgery Aborted Surgery performed after 2 weeks under PB
Sethi et al., 2020 [7]	India	Case Report	1 1 (100%)	65 Male	Left	N/a	HTN ASA: 2	8 mL of 1:1 mixture of 2% lignocaine and 0.5% bupivacaine with 25 IU/mL of hyaluronidase 25 mm, 24 G 2 First Injection: 5 mL Inferotemporal, transconjunctival injection inserted at the junction of the medial two-thirds and the lateral third of the lower eyelid Second Injection: 3 mL: medial conal space	5 min after the second injection	Respiratory Arrest Bradicardia Hypotension	Respiratory Arrest: Ventilatory assistance: Intubation Bradicardia: IV Atropine 0.6 mg Hypotension: Ringer Lactate infusion	Extubation and Surgery Performed
Basu et al., 2021 [6]	India	Case Report	1 1 (100%)	55 Male	Right	N/a	None ASA: N/a	7 mL of 2% lignocaine (mixture of lidocaine 2%, with hyaluronidase 50 IU/mL, and adrenaline 1:200,000) 25 mm, 24 G 1 N/a	3 min after the PB	Respiratory Arrest Hypotension Seizure	Respiratory Arrest: Ventilatory assistance: Intubation Seizure: IV midazolam (2.0 mg)	Surgery Aborted Surgery performed after 3 months

Abbreviations: N/a: not applicable; BSA: Brainstem Anesthesia; n: Number; PB: Peribulbar block; ASA: American Society of Anaesthesiologists; IU: International Unit; IV: Intravenous; mm: millimeter; GA: General Anesthesia; CT: computed tomography; CPR: Cardiopulmonary resuscitation; ICU: Intensive Care Unit; min: minute; mmHg: millimeter of mercury; mg: milligram; mL: milliliter; G: Gauge; DM: diabetes Mellitus; HTN: Hypertension.

## Supplementary Materials

The following supporting information can be downloaded at https://www.mdpi.com/article/10.3390/jcm13216572/s1, Supplementary Material S1: Detailed Search Strategy. Supplementary Material S2: PRISMA Checklist. Supplementary Material S3 (Table S1: Newcastle–Ottawa Scale for Critical Appraisal of Cross-Sectional Studies; Table S2: Joanna Briggs Institute Critical Appraisal Checklist for Case Reports; Table S3: GRADE assessment).
