# Supplementary material for: Brainstem Anesthesia and Cardiac Arrest Following Peribulbar Block: A Case Report and Systematic Review of the Literature
Source: J Clin Med. 2024 Nov 1;13(21):6572. doi: 10.3390/jcm13216572 (PMC11546325; doi:10.3390/jcm13216572)
Supplement: Supplementary file 1 [file jcm-13-06572-s001.zip › jcm-3276762-Supplementary Material S1.pdf]

Detailed Search Strategy

PUBMED:
Free Text and MESH
Search updated on 1 June 2024

Query: (("peribulbar"[All Fields] AND ("anaesthesia"[All Fields] OR "anesthesia"[MeSH Terms] OR "anesthesia"[All Fields] OR "anaesthesias"[All Fields] OR "anesthesias"[All Fields])) OR ("peribulbar"[All Fields] AND ("block"[All Fields] OR "blocked"[All Fields] OR "blocking"[All Fields] OR "blockings"[All Fields] OR "blocks"[All Fields]))) AND (("brain stem"[MeSH Terms] OR ("brain"[All Fields] AND "stem"[All Fields]) OR "brain stem"[All Fields] OR "brainstem"[All Fields] OR "brainstems"[All Fields] OR "brainstem s"[All Fields]) AND ("anaesthesia"[All Fields] OR "anesthesia"[MeSH Terms] OR "anesthesia"[All Fields] OR "anaesthesias"[All Fields] OR "anesthesias"[All Fields]))

#	Search Term	No. Results
1	"Brainstem Anesthesia"	4601
2	"Peribulbar Anesthesia" OR "Peribulbar Block"	855
4	1 AND 2	14

EMBASE

Free Text
Search updated on 1 June 2024

Mapping options enabled:
- map to preferred term in Emtree
- search also as free text in all fields
- explode using narrower Emtree terms
- search as broadly as possible

#	Search Term	No. of Results
1	(("Brainstem Anesthesia " AND "Peribulbar Anesthesia" OR "Peribulbar Block"))	21

SCOPUS

Free Text
Search updated on 1 June 2024

#	Search Term	No. of Results
1	(TITLE-ABS-KEY (Brainstem Anesthesia) AND TITLE-ABS-KEY (Peribulbar AND Anesthesia) OR TITLE-ABS-KEY (Peribulbar AND Block))	18
