# Supplementary material for: Brainstem Anesthesia and Cardiac Arrest Following Peribulbar Block: A Case Report and Systematic Review of the Literature
Source: J Clin Med. 2024 Nov 1;13(21):6572. doi: 10.3390/jcm13216572 (PMC11546325; doi:10.3390/jcm13216572)
Supplement: Supplementary file 1 [file jcm-13-06572-s001.zip › jcm-3276762-Supplementary Material S3.pdf]

**Table S1: Newcastle–Ottawa Scale for Critical Appraisal of Cross-Sectional Studies**

| | | Selection | | | | Comparability | Outcome | | |
|---|---|---|---|---|---|---|---|---|---|
| **Source** | **Study design** | **Representativeness of the sample** | **Sample size** | **Non-respondents** | **Ascertainment of the exposure:** | | **Assessment of outcome** | **Statistical test** | **Total score** |
| Eke et al., 2007 [1] | Cross-Sectional | +1 (b) | +1 (a) | +1 (b) | +1 (a) | +1 (a) | +2 (a) | +1 (a) | 8/10 |

**Representativeness of the sample**: a) Truly representative of the average in the target population. * b) Somewhat representative of the average in the target population; * c) Selected group of users d) No description of the sampling strategy; **Sample size**: a) Justified and satisfactory. * b) Not justified; **Non-respondents**: a) Comparability between respondents and non-respondents characteristics is established, and the response rate is satisfactory. * b) The response rate is unsatisfactory, or the comparability between respondents and nonrespondents is unsatisfactory. c) No description of the response rate or the characteristics of the responders and the nonresponders.; Ascertainment of the exposure: a) Validated measurement tool. * b) Non-validated measurement tool, but the tool is available or described. c) No description of the measurement tool.;
**Comparability: (Maximum 2 stars)** 1) The subjects in different outcome groups are comparable, based on the study design or analysis. Confounding factors are controlled. a) Data/ results adjusted for relevant predictors/risk factors/confounders ** b. Data/results not adjusted for all relevant confounders/risk factors/information not provided.
**Outcome (Maximum 2 stars):** 1) Assessment of the outcome: a) Independent blind assessment *; b) Record linkage *; c) Self report; d) No description. 2) Statistical test: a) The statistical test used to analyze the data is clearly described and appropriate, and the measurement of the association is presented, including confidence intervals and the probability level (p value) *; b) The statistical test is not appropriate, not described or incomplete.

*Cross-sectional Studies: Very Good Studies: 9-10 points Good Studies: 7-8 points Satisfactory Studies: 5-6 points Unsatisfactory Studies: 0 to 4 points*

**Table S2: Joanna Briggs Institute Critical Appraisal Checklist for Case Reports**

| Source | Were patient's demographic characteristics clearly described? | Was the patient's history clearly described and presented as a timeline? | Was the current clinical condition of the patient on presentation clearly described? | Were diagnostic tests or assessment methods and the results clearly described? | Was the intervention(s) or treatment procedure(s) clearly described? | Was the post-intervention clinical condition clearly described? | Were adverse events (harms) or unanticipated events identified and described? | Does the case report provide takeaway lessons? |
|---|---|---|---|---|---|---|---|---|
| Edge et al. 1995 [2] | yes | yes | yes | yes | yes | yes | no | yes |
| Gomez et al. 1997 [3] | yes | yes | yes | yes | yes | yes | no | yes |
| Carneiro et al. 2007 [4] | yes | yes | yes | yes | yes | yes | no | yes |
| Jaichandran et al., 2013 [5] | yes | yes | yes | yes | yes | yes | no | yes |
| Palte et al., 2017 [5] | yes | yes | yes | yes | yes | yes | no | yes |
| Kazancıoğlu, 2017 [7] | yes | yes | yes | yes | yes | yes | no | yes |
| Vohra et al., 2019 [8] | yes | yes | yes | yes | yes | yes | no | yes |
| Tayab et al., 2019 [9] | yes | yes | yes | yes | yes | yes | no | yes |
| Sethi et al., 2020 [10] | yes | yes | yes | yes | yes | yes | no | yes |
| Basu et al., 2021[11] | yes | yes | yes | yes | yes | yes | no | yes |

**Table S3: GRADE assessment**

| Certainty assessment | | | | | | | | | Certainty |
|---|---|---|---|---|---|---|---|---|---|
| Outcome | Studies (n) | Study design | Risk of bias | Inconsistency[a] | Indirectness[d] | Imprecision[c] | Publication bias[b] | Patients (n) | |
| Prevalence of BSA following PB [1-11] | 11 | Observational studies and Case Reports | serious | serious | not serious | serious | Undetected | 15 | ⊕⊕◯◯ Low |

**CI:** Confidence interval; **MD:** Mean difference; **OR:** Odds ratio; [a] Substantial heterogeneity $I^2 > 60\%$ (serious) or >90% (very serious); [b] Strongly suspected if funnel plot suggestive of publication bias or lack of small studies and negative effects; [c] serious if the total number of events is less than 300, CIs overlap or non clinically significant effect; [d] Serious indirectness refer to variation of outcome measure or definition across studies

## References


1. Eke, T.; Thompson, J.R. Serious Complications of Local Anaesthesia for Cataract Surgery: A 1 Year National Survey in the United Kingdom. *Br. J. Ophthalmol.* **2007**, *91*, 470–475.
2. Edge, K.R.; Davis, A. Brainstem Anaesthesia Following a Peribulbar Block for Eye Surgery. *Anaesth. Intensive Care* **1995**, *23*, 219–221.
3. Gomez, R.; Andrade, L.O.F.; Rezende Costa, J.R.; Santiago Gomez, R. Brainstem Anaesthesia after Peribulbar Anaesthesia. *Can. J. Anaesth.* **1997**, *44*, 7–732.
4. Carneiro, H.M.; Oliveira, B.; Ávila, M.P.; Neto, O.A. Anestesia Do Tronco Encefálico Após Bloqueio Retrobulbar Extraconal. É Possível Evitar? Relato de Caso. *Rev. Bras. Anestesiol.* **2007**, *57*, 391–400.
5. Jaichandran, V.V.; Nair, A.G.; Gandhi, R.A.; Prateeba-Devi, N. Brainstem Anesthesia Presenting as Contralateral Third Nerve Palsy Following Peribulbar Anesthesia for Cataract Surgery. *Acta Anaesthesiol. Taiwan* **2013**, *51*, 135–136.
6. Palte, H.D.; Hoa, D.P.; Pavon Canseco, A. Surdity in the OR: An Unusual Case of Brainstem Anesthesia. *Case Rep. Anesthesiol.* **2017**, *2017*, 4645381.
7. Kazancıoğlu, L.; Batçık, Ş.; Kazdal, H.; Şen, A.; Gediz, B.Ş.; Erdivanlı, B. Complication of Peribulbar Block: Brainstem Anaesthesia. *Turk. J. Anaesthesiol. Reanim.* **2017**, *45*, 231.
8. Vohra, S.B.; Nagi, H. Brainstem Anaesthesia Following Single Medial Canthal Peribulbar Block: A Case Report and Review. *Trends Anaesth. Crit. Care* **2019**, *28*, 1–5.
9. Tayab, S.; Paul, S.; Sarma, P. Potentially Life-Threatening Consequences of Peribulbar Anesthesia. *TNOA J. Ophthalmic Sci. Res.* **2019**, *57*, 231.
10. Sethi, S.; Sethi, M. Brainstem Anesthesia: An Infrequent yet Catastrophic Complication of Peribulbar Block. *Int. J. Dent. Med. Sci. Res.* **2020**, *2*, 286–289.
11. Basu, C.; Basak, S. Brainstem Anesthesia: A Rare Complication after Peribulbar Anesthesia. *Indian. J. Case Rep.* **2021**, *7*, 47–49.
